# Early Adolescent Cognitive Gains Are Marked by Increased Sleep EEG Coherence

**DOI:** 10.1371/journal.pone.0106847

**Published:** 2014-09-10

**Authors:** Leila Tarokh, Mary A. Carskadon, Peter Achermann

**Affiliations:** 1 University Hospital of Child and Adolescent Psychiatry and Psychotherapy, University of Bern, Bern, Switzerland; 2 Institute of Pharmacology and Toxicology, University of Zurich, Zurich, Switzerland; 3 Department of Psychiatry and Human Behavior, Alpert Medical School of Brown University, Providence, Rhode Island, United States of America; 4 Centre for Sleep Research, School of Psychology, Social Work and Social Policy, University of South Australia, Adelaide, Australia; 5 Zurich Center for Integrative Human Physiology, University of Zurich, Zurich, Switzerland; 6 Neuroscience Center, University and ETH Zurich, Zurich, Switzerland; 7 Zurich Center for Interdisciplinary Sleep Research, University of Zurich, Zurich, Switzerland; Hôpital du Sacré-Coeur de Montréal, Canada

## Abstract

Although the increases in cognitive capacities of adolescent humans are concurrent with significant cortical restructuring, functional associations between these phenomena are unclear. We examined the association between cortical development, as measured by the sleep EEG, and cognitive performance in a sample of 9/10 year olds followed up 1 to 3 years later. Our cognitive measures included a response inhibition task (Stroop), an executive control task (Trail Making), and a verbal fluency task (FAS). We correlated sleep EEG measures of power and intra-hemispheric coherence at the initial assessment with performance at that assessment. In addition we correlated the rate of change across assessments in sleep EEG measures with the rate of change in performance. We found no correlation between sleep EEG power and performance on cognitive tasks for the initial assessment. In contrast, we found a significant correlation of the *rate of change* in intra-hemispheric coherence for the sigma band (11 to 16 Hz) with *rate of change* in performance on the Stroop (r = 0.61; p<0.02) and Trail Making (r = −0.51; p<0.02) but no association for the FAS. Thus, plastic changes in connectivity (i.e., sleep EEG coherence) were associated with improvement in complex cognitive function.

## Introduction

Cognitive capacities blossom during adolescence, setting the stage for adult cognitive proficiency [1]. The cortical restructuring (i.e., reduced cortical grey matter volume and increased cortical white matter volume [2]) attendant to adolescent development likely bolsters expanded cognitive capabilities, although the specifics of this association are unclear. Longitudinal studies, which measure both cortical volume and cognitive abilities, can uniquely address whether the changes in grey and white matter volume underlie the adolescent expansion of cognitive capacities. A further strength of longitudinal studies is that they control for the large inter-individual variability observed in the rate at which cortical grey/white matter develop [3]. Thus far, three longitudinal MRI studies of adolescents have examined the association between cortical volume and general cognitive abilities (e.g., Intelligence Quotient) or executive function, finding that the rate of change in cortical thickness in specific brain regions was associated with cognitive function [4]–[6].

Structural maturation of the brain can be identified not only with MRI but is also, to an extent, reflected in the EEG. For example, a recent study found an association between sleep EEG power in the delta band (1 to 4.5 Hz) and grey matter volume in the same individuals, with both measures diminished in older compared to younger adolescents [7]. On the other hand, increased white matter volume may be reflected in increased correlation of EEG activity, i.e. *coherence*, from distal electrode placements [8]. Coherence is thought to be a measure of structural and functional connectivity, because strong associations between the activities of distal neural populations provides evidence that these regions are connected or are driven by a common source. Similar to white matter volume, sleep EEG coherence within a hemisphere (i.e., intra-hemispheric coherence) increases in a linear manner in early childhood (ages 2 to 5 years) [9] and across adolescence (ages 9 to 23 years) [10]. In both children and adolescents, the increases in sleep EEG coherence with age has been found in rapid eye movement (REM) and non-REM (NREM) sleep – two very different cortical milieus – further evidence that structural changes to the brain underlie the observed changes in sleep EEG coherence.

**Figure 1:**
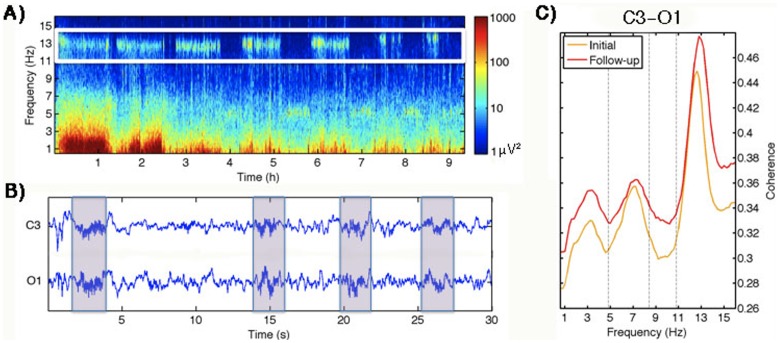
This figure demonstrates the manifestation of sleep spindles in the sleep EEG. A) Time-frequency spectrogram for a baseline night of a 12-year-old boy with the spindle (sigma) band highlighted by a white box. Time (h) is on the x-axis and frequency is on the y-axis. Large power values are shown in warm colors, while cool color depict lower power. B) Thirty-second EEG signal of a left central (C3/A2) and left occipital (O1/A2) derivation with spindles highlighted with boxes. C) All-night intra-hemispheric coherence spectrum (C3/A2 and O1/A2) averaged across all subjects at the initial (ages 9/10 years) and follow-up (ages 11–13 years) assessments.

In addition to reflecting brain maturation, the sleep EEG provides the added benefit of measuring brain activity unperturbed by external stimuli. For example, sleep spindles are transient oscillations between 11 to 16 Hz generated during NREM sleep through long-range thalamocortical loops and likely depend on both structural and functional connectivity (Figure 1b) [11]. These oscillations can, in part, be captured in the sleep EEG power and coherence spectra as a peak in the frequency range corresponding to sleep spindles (i.e., 11 to 16 Hz). Previous studies have shown power in the sigma band is dominated by organized spindles [12]–[14], although the absence of a perfect correlation implies that sigma band power comprises more than spindles alone.

We analyzed sleep EEG data collected in a longitudinal study to examine associations of sleep EEG measures of power and coherence with cognitive performance in 9 and 10 year old children who had overnight polysomnographic recordings on the night after cognitive tests. Since successful performance on a cognitive task requires integrity of structural and functional connectivity between distal brain regions, we speculate that sleep spindles may be a probe of such connectivity. Furthermore, a large literature points to associations between spindles and cognitive function. This association appears to be non-specific and has been seen for overnight learning of declarative and procedural memory, as well as intelligence (i.e., IQ; reviewed in [15]). Thus, we hypothesize that power and coherence in the frequency band corresponding to sleep spindles (sigma; 11 to 16 Hz) will be associated with performance on neuropsychological tests. More specifically, we expect the developmental decline in sigma EEG power will be negatively associated with improved performance, while the maturational increase in sigma band coherence will show a positive correlation with performance.

## Materials and Methods

Data were collected from twenty healthy children when they were 9 or 10 years old (mean age = 10.1±0.56 years; 7 female; one left handed) and again 1 to 3 years later (mean age = 12.5±0.76 years). Children were excluded from the study if they had a current or chronic illness, evidence of a learning disability, personal history of a sleep disorder or a family history of a psychiatric disorder (other than alcohol dependence/abuse). Furthermore, children with a pattern of chronic insufficient sleep or excessive daytime sleepiness as indicated by sleeping for less than 9 hours nightly, or taking naps more than twice per week were excluded. The Lifespan Institutional Review Board approved all procedures and written consent from parents and verbal assent from children was obtained prior to enrollment in the study. The protocol at each assessment was identical, ensuring the same amount of sleep at both time points (mean in-lab sleep time at visit 1 = 568±21 min; mean in-lab sleep time at visit 2 = 560±20 min; paired t-test t(19) = 1.55; p = 0.14). Participants spent at least one week on a consistent sleep/wake schedule that ensured a minimum of 10 h time in bed before two consecutive nights sleeping in the laboratory. Cognitive testing took place in the laboratory [10∶00–11∶00 am] on the day between the two overnight sleep recordings. Sleep EEG data from the second night were used in the analysis. Participants slept in individual darkened bedrooms while polysomnography (PSG) was recorded. PSG recordings included two central (C3/A2 and C4/A1) and two occipital (O1/A2 and O2/A1) EEG derivations, right and left electrooculogram, electromyogram, and electrocardiogram. Sleep data were visually scored in 30-s epochs using the criteria of Rechtschaffen and Kales [16]. Artifacts were rejected using a semi-automatic procedure [17].

All-night power and coherence spectra were calculated separately for rapid eye REM and NREM sleep across the night using MATLAB and its signal analysis and statistics toolbox (MathWorks Inc, Nantick, MA, USA). Despite the predominance of sleep spindles during stage 2 sleep, the entirety of NREM sleep (stages 2, 3, and 4) was used to account for slow wave sleep decline and stage 2 sleep increase with age [18]. Sleep EEG power and coherence spectra were calculated for each 30-s epoch (Hanning window, average of six 5-s epochs), resulting in a frequency resolution of 0.2 Hz (for details see [18]; [10]). A time by frequency spectrogram, which shows power across a night as a function of frequency for one exemplary subject, is provided in Figure 1a. Coherence was defined as the squared cross-spectrum between signals divided by the product of the auto-spectra of each signal (magnitude squared coherence). It comprises a correlation measure in the frequency domain comparing the relation between two signals (i.e. EEG derivations). Coherence values range from 0 to 1, where 1 indicates full correlation (synchronization) between two signals at a given frequency, while a value near zero suggests that the signals are unrelated. Only coherence within a hemisphere (intra-hemispheric) was used in this study, as previous findings from the same data set indicate a maturational increase in intra- but not inter- hemispheric coherence [12]. Furthermore, in order to reduce multiple comparisons and since both hemispheres show a linear increase in intra-hemispheric coherence, coherence was evaluated using an average of intra-hemispheric coherence for both hemispheres in each participant. Thus, coherence was computed as the mean intra-hemispheric coherence of derivations in the left (C3/A2 and O1/A2) and right (C4/A1 and O2/A1) hemispheres. Coherence spectra averaged across subjects between derivations C3/A2 and O1/A2 are shown in Figure 1c for both assessments. In addition to examining power and coherence in the sigma band (11 to 16 Hz), exploratory analyses also examined associations in three other conventional frequency bands: delta (0.6 to 4.8 Hz), theta (5 to 8.4 Hz), and alpha (8.6 to 10.8 Hz). A rate of change measure was computed for each band for power and coherence. This measure was computed as the power/coherence at the follow-up assessment minus the initial assessment divided by the number of months between assessments.

Neuropsychological testing included the Stroop [19], Trail Making [20], and FAS [21] tasks. The Stroop task is a measure of response inhibition, selective attention, and cognitive flexibility. We chose the incongruent condition of the Stroop task in which participants were asked to inhibit the impulse to read the color-word and to name the color the word is written in (e.g., the word RED in blue ink = blue correct response). Our outcome variable was the number of correct responses the participants provided in 45 seconds. The Trail Making Test (Trails) measures visual attention and task switching. Part A of this task consists of drawing a line between consecutive numbers, while Part B requires participants to switch between numbers and letters. Our outcome variable consisted of the time it took to complete Part B minus the time needed for Part A. Our third cognitive task, the FAS, is a measure of verbal fluency and requires participants to name as many words beginning with an “F”, “A”, and “S” with 60 seconds allotted for each category. Our outcome variable was the total number of words participants articulated across categories. Similar to sleep EEG parameters, we examined performance at the initial assessment as well as rate of change. As with the EEG variables, rate of change was defined for each participant as performance score at the follow-up assessment minus the initial assessment, divided by the number of months between assessments.

Robust regressions (Matlab function robustfit; default setting) were calculated between cognitive task performance and sleep EEG power/coherence for each frequency band at the initial assessment, as well as for our rate of change measures of each domain. To test our *a priori* hypothesis that sigma band power/coherence at time one and rate of change is correlated with neuropsychological tests scores, we set alpha at 0.05. For our exploratory analysis of the association between other frequency bands and neuropsychological performance with a sample size of twenty a correlation coefficient above 0.6 was considered statistically significant [22].

## Results

We observed improved performance of the Stroop (t(19) = 6.44; p<0.0001), Trails (t(19) = 2.24; p<0.037), and FAS (t(19) = −3.27; p<0.004) tasks across assessments (data can be found in Data S1).

Developmental changes in sleep EEG power and coherence have previously been described for this data set [10], [18]. Briefly, we found a frequency-independent maturational increase in intra-hemispheric sleep EEG coherence (Figure 1) and a decline in power (Figure S1) for both NREM and REM sleep.

Neither sleep EEG power nor coherence was correlated with task performance at the initial assessment for any frequency band during NREM or REM sleep. We then examined whether the rate of decline in sleep EEG power and rate of increase in intra-hemispheric coherence were correlated within each frequency band. Rates of change in these variables were not correlated for any frequency band. This finding indicates that despite concurrent developmentally-mediated brain cortical restructuring, the decline in EEG power (reflecting grey matter volume) and increase in coherence (perhaps indexing white matter) develop independently in this age range.

**Figure 2:**
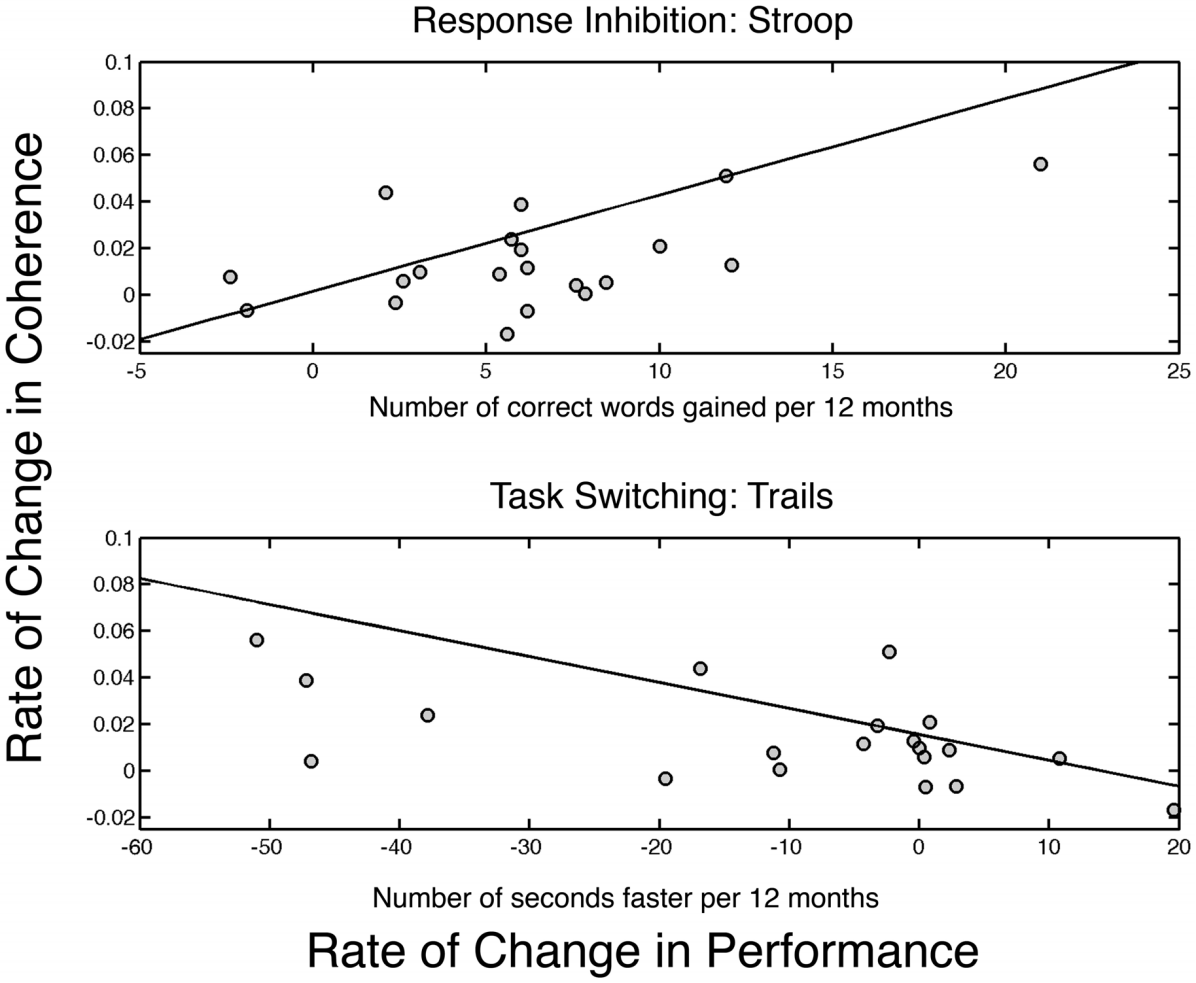
Rate of change in performance per year is plotted on the x-axis, while rate of change in intra-hemispheric sigma band coherence per year is plotted on the y-axis. The top plot shows the results for the Stroop task in which the number of words correctly identified in the incongruent condition increases with age. The bottom shows the rate of change in the Trails task in which the time to completion is measured. The line represents the robust fit to the data. Thus, improved performance is indicated by a larger value for the Stroop and smaller value for the Trails. Lines represent robust regression fit to the data.

We then probed whether rates of change in sleep EEG power or in intra-hemispheric coherence were related to cognitive performance gains. We found a significant association for rate of change in intra-hemispheric coherence and performance only in the sigma band (11 to 16 Hz) for NREM sleep. The finding was consistent for the Stroop and Trails tasks: Stroop task (r = 0.61; p = 0.019); Trails tasks (r = −0.51; p = 0.012). No association was found for the FAS (r = −0.23; p = 0.13). Figure 2 shows the correlation between the rate of change in sigma band coherence and rate of change for performance on the Stroop task (measured in words/year) in the top panel, while the bottom panel is the results of the Trails task (measured in seconds/year). No associations emerged for NREM and REM sleep EEG power with performance gains.

## Discussion

We demonstrated here in early adolescents that rate of change in performance for complex cognitive tasks were uniquely correlated to the rate of increase in intra-hemispheric sleep EEG coherence in the sigma band. Sigma band power is associated with sleep spindles and is a prominent feature of the NREM sleep EEG signal (see Figure 1a). Sleep spindle-like activity emerges in children as young as two months [23], and sleep spindles are found across the lifespan, though with reduced integrity and rate of occurrence in old age and with dementia [24]. The generation of sleep spindles requires both local circuits and long-range thalamocortical loops [11]. Thus, sleep spindles – that is sigma band EEG activity in NREM sleep – serve as a hallmark of the brain’s structural integrity as well as a possible indication of its functional efficiency.

Several lines of research have found links between sleep spindles, sigma band power, and cognitive function. For example, sleep EEG sigma power and sleep spindles *per se* have been related to overnight learning and memory [24]. Sleep EEG sigma band power has also been proposed as a marker of intellectual ability with a cross-sectional study of children between the ages of 9 and 12 years finding a positive correlation between IQ measures and sigma band power [25]. We add to the existing literature by showing that measures of executive function that are largely independent of IQ [26] are associated with sleep EEG sigma coherence in a dynamic manner. Similar to the MRI studies [5], we find that the rate of increase, rather than coherence at a single time point, is associated with improved task performance.

We conjecture that the change in coherence indicates increased intra-hemispheric thalamocortical connectivity in these early adolescents. Furthermore, this “improved” connectivity may be uniquely reflected in sleep spindle activity, which is a combined measure of structural and functional connectivity.

Contrary to our hypothesis we found no association between sleep EEG power and cognitive performance for either concurrent or rate of change assessments. Although the absence of such an association may reflect dissociation between sleep EEG power and cognitive function, this outcome may also arise from the limited spatial resolution due to the few derivations investigated. Indeed, executive functioning is often associated with the (pre)frontal cortex, a region that was not sampled in this study.

We conclude that our data provide evidence of plastic changes in early adolescent brain connectivity, and that the rate of this neural maturation supports gains in complex cognitive performance.

## Supporting Information

Figure S1 **Power density spectra.** Subject average power density spectra for the initial (green) and follow-up (blue) assessments for derivation C3/A2.
(TIF)

Data S1 **Cognitive performance and coherence data at both assessments.** Excel file with the data used in the analysis. Columns are: Time between assessment (in years); Gender (M = Male and F = Female); Stroop Initial and Follow-up (the number of words correctly identified on the Stroop at the initial assessment and follow-up assessments, respectively); Trails the time to complete the trails (Part B minus Part A) measured in seconds; NREM sigma Intrahemispheric Coherence at the initial and follow-up assessments.
(XLSX)
