# Supplementary figures and images for: Early Adolescent Cognitive Gains Are Marked by Increased Sleep EEG Coherence
Source: PLoS One. 2014 Sep 10;9(9):e106847. doi: 10.1371/journal.pone.0106847 (PMC4160237; doi:10.1371/journal.pone.0106847)

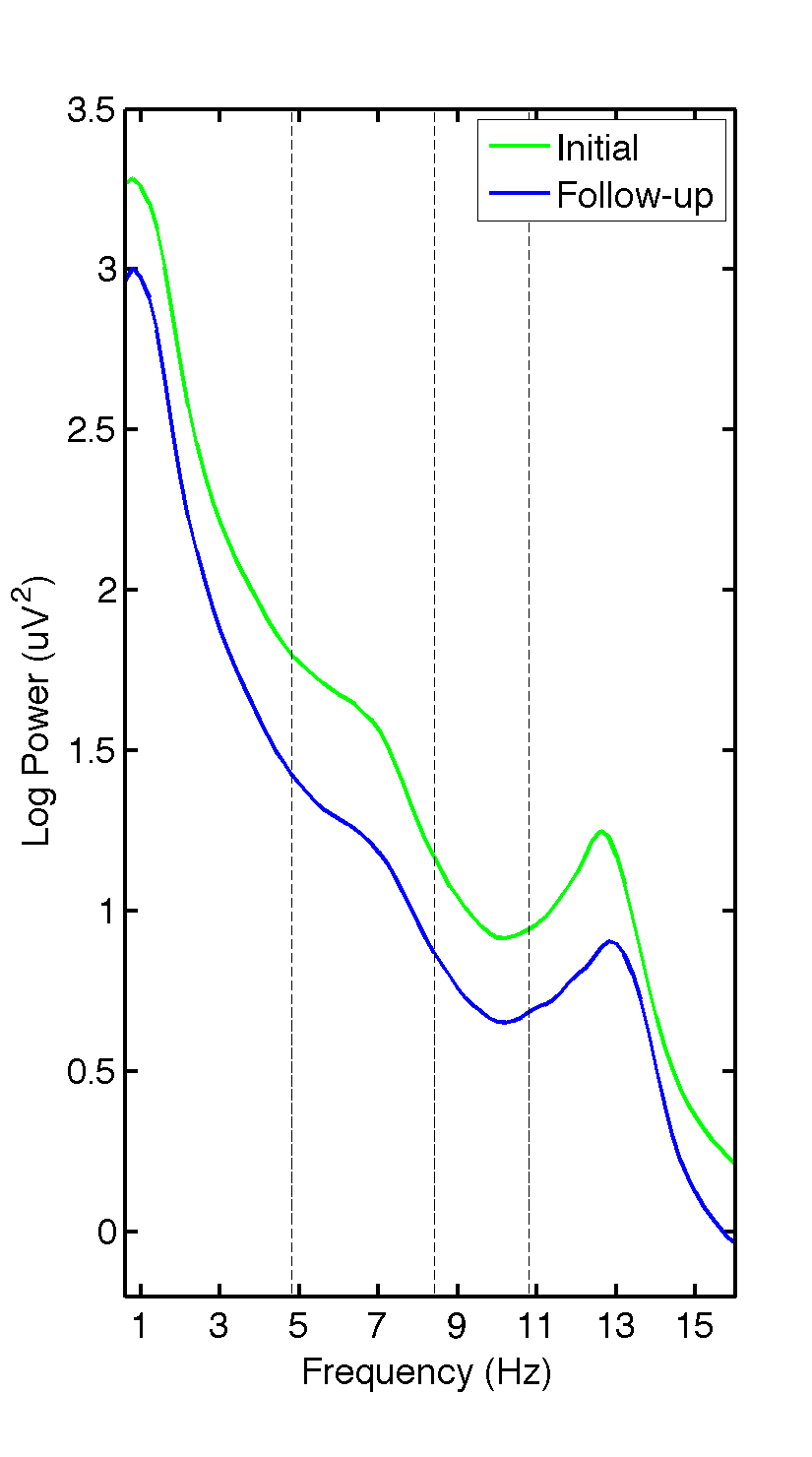

Supplement: Figure S1 — Power density spectra. Subject average power density spectra for the initial (green) and follow-up (blue) assessments for derivation C3/A2. (TIF) [file pone.0106847.s001.tif]
